# Implementation and Scale-Up of the Standard Days Method of Family Planning: A Landscape Analysis

**DOI:** 10.9745/GHSP-D-19-00287

**Published:** 2020-03-30

**Authors:** Julianne Weis, Mario Festin

**Affiliations:** a United States Agency for International Development, Washington, DC, USA.; b World Health Organization, Geneva, Switzerland.

## Abstract

The Standard Days Method (SDM), a modern fertility awareness-based family planning method, has been introduced in 30 countries since its development in 2001. It is still unclear to what extent the SDM was mainstreamed within the family planning method mix, particularly in low- and middle-income country (LMIC) settings, where the SDM had been introduced by donors and implementing partners. This review of implementation science publications on the SDM in LMICs first looked at community pilot studies of the SDM to determine the acceptability of the method; correct use and efficacy rates; demographics of users; and changes to contraceptive prevalence rates and family planning behaviors, especially among men and couples. Then, we examined the status of the SDM in the 16 countries that had attempted to scale up the method within national family planning protocols, training, and service delivery. At the community level, evidence demonstrated a high level of acceptability of the method; efficacy rates comparable to the initial clinical trials; diversity in demographic characteristics of users, including first-time or recently discontinued users of family planning; increased male engagement in family planning; and improved couple's communication. Nationally, few countries had scaled up the SDM due to uneven stakeholder engagement, lackluster political will, and competing resource priorities. Results of this review could help policy makers determine the added value of the SDM in the contraceptive method mix and identify potential barriers to its implementation moving forward.

## Abstract (precis)

Pilot introductions of the Standard Days Method (SDM) of family planning demonstrated its potential to meet unmet contraceptive needs in key populations, strengthen male involvement, and increase overall contraceptive uptake. Few countries had implemented national scale-up due to barriers, such as competing resource priorities and uneven stakeholder engagement. Demand-side user barriers, including insufficient fertility awareness knowledge, were also constraints. Policy makers should determine the SDM's added value to the contraceptive method mix and identify potential barriers to its implementation.

## INTRODUCTION

The Standard Days Method (SDM) is a fertility awareness-based family planning method that identifies a 12-day fertile window during which women with regular menstrual cycles (26–32 days long) should abstain from sex or use a barrier method to prevent pregnancy. SDM is limited to women with regular menstrual cycles of 26–32 days, which applies to an estimated 50%–60% of women of reproductive age, though contraindications, including recent pregnancy and breastfeeding, can also affect cycle regularity and eligibility for the method.^1^

First developed and tested in 2001 by the Institute for Reproductive Health (IRH), the SDM was introduced with “CycleBeads,” a string of different colored beads that each represent 1 day in the menstrual cycle, as a visual tracking tool to facilitate correct use of the method. Brown beads indicate nonfertile days, and white beads indicate fertile days when the user should abstain from sex or use a barrier method. The user moves a small rubber ring along the CycleBeads string each day to track their fertility. In 2012, the free iCycleBeads app was introduced and piloted as a digital version of the CycleBeads for download on a mobile device.

The SDM is 95% effective in perfect use and 88% effective in typical use.^2^ Classified as a modern method of family planning by the World Health Organization, U.S. Centers for Disease Control and Prevention, and other international health organizations, the SDM has been introduced in 30 countries globally in an effort to expand contraceptive method choice.

Although the SDM was introduced largely in pilot programs by nongovernmental organizations (NGOs) and donor agencies that supported ministries of health (MOHs), it remains unclear to what extent the SDM was implemented or scaled up at the national level within health systems. Reported use of the SDM captured in Demographic and Health Surveys remained less than 1% across countries where it was introduced. Reported knowledge of the SDM also varied considerably in Demographic and Health Surveys, from less than 1% in India to 82% in Rwanda, with a median of 26% (STATcompiler, ICF International, 2012, Washington, DC).

**Figure:**
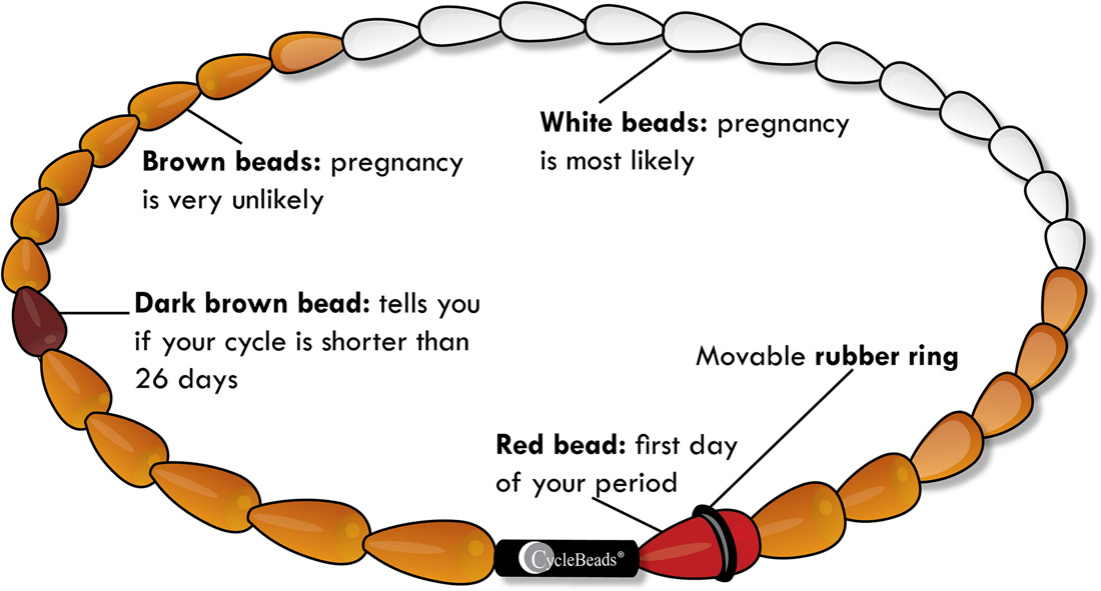
CycleBeads have colored beads that each correspond to 1 menstrual cycle day. © 2019/Institute for Reproductive Health

**Figure:**
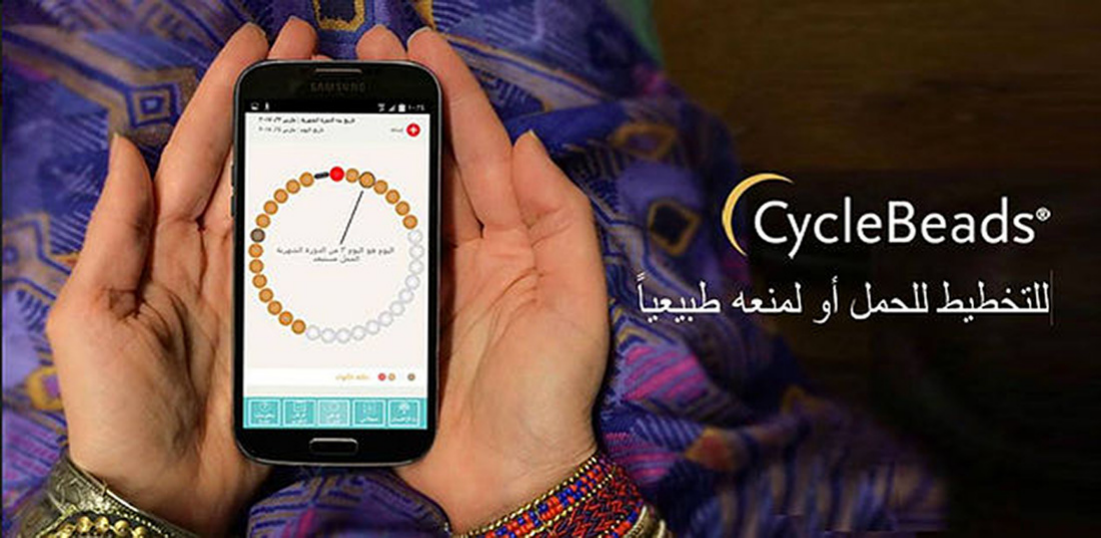
The iCycleBeads mobile device app in Arabic. © 2019/Cycle Technologies

This landscape analysis of implementation studies of the SDM intended to answer the following key questions:

- What happened when the SDM was first introduced in low- and middle-income country (LMIC) settings?
- Was the SDM an effective and feasible method of family planning for users?
- What was the status of the SDM implementation and scale-up at national levels in LMICs?

This analysis focused on SDM implementation in low-resource settings and user-based outcomes, including users' approval of the method, continuation rates, and efficacy, as well as the impact of attempts to institutionalize the SDM within the family planning method mix at the national level. This landscaping review examined implementation science of the SDM specifically in LMIC settings from a user perspective and complemented other reviews on method efficacy.^3^ This analysis helped examine the barriers and enablers to the SDM's introduction at both community and national levels and could help inform broader policy on fertility awareness-based methods in LMICs moving forward.

## DATA AND METHODS

To complete the landscape analysis, we completed database searches in both PubMed and Google Scholar using the search terms “Standard Days Method + family planning + implementation.” We limited the search results to publications from 2000 to 2019 because of the SDM's recent development as a fertility awareness-based method.

PubMed recovered 10,489 results, and Google Scholar found 16,300 results. After scrutinizing the titles of all 26,789 results, 165 studies were included for further screening of abstracts. All 165 studies addressed some aspect of the SDM of family planning, whereas the other search results were unrelated to the SDM specifically. Of these, 97 studies were duplicates in both PubMed and Google Scholar search results; therefore, 58 studies were selected for further review from Google Scholar and 10 from PubMed (Figure).

**FIGURE:**
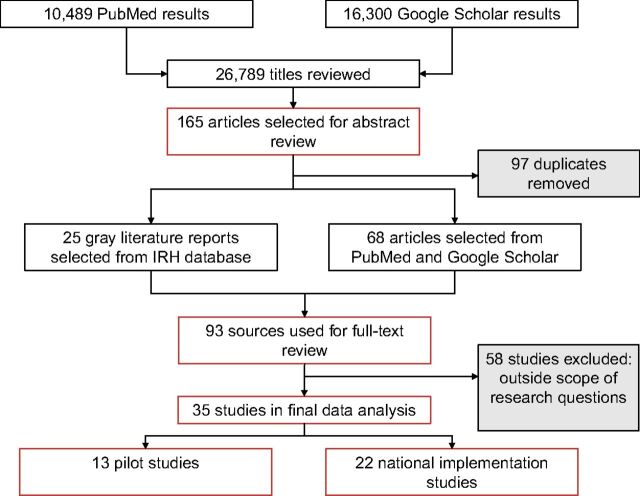
Methodology of Landscape Analysis Review of SDM Studies Abbreviations: IRH, Institute for Reproductive Health; SDM, Standard Days Method.

In addition to conducting database searches, we searched for gray literature reports on the IRH website. Because of IRH's long history in developing and implementing the SDM, we understood that their program materials had significant evidence on the status of the SDM in LMICs, especially in terms of nationalization and scale-up. This search yielded an additional 25 gray literature reports, including annual reports, project briefs, and evaluation materials.

We completed a full-text analysis of the 93 sources identified in both database and IRH searches. In completing this analysis, we excluded 58 studies that did not provide answers to our initial research questions on user-based outcomes of the SDM implementation and status in national family planning systems in LMICs. Ten studies were excluded because they did not explicitly study the SDM but merely mentioned the SDM in conjunction with other family planning methods. We excluded 12 studies that provided general commentary on the SDM as an addition to the family planning method mix but did not provide new evidence of the SDM's implementation. Five studies were excluded because they were efficacy studies of the SDM, so they relied on only 1 data point (pregnancy rate) in a clinical trial setting. We were interested in examining other outcomes of the SDM and its feasibility as a method when implemented in more routine family planning practice. Sixteen studies were excluded because they were not conducted in an LMIC setting. We excluded 9 studies on the social marketing and provider-side issues of the SDM implementation because these were outside the scope of our research questions. Studies that examined provider-based outcomes, including training, feasibility of counseling, and marketing methodologies, warrant a separate rigorous analysis and review. Lastly, 6 studies were excluded because they did not provide sufficient data on user-based outcomes of the SDM implementation. These studies did not study the SDM explicitly (included user numbers along a range of other family planning methods introduced in a family planning project) and/or included only 1 data point (e.g., pregnancy rate) and included nothing about correct use, satisfaction with method, or other user-based outcomes. Many of the studies that were excluded from full data analysis informed the discussion section of our review.

Using these exclusion criteria, 35 studies were included for final, in-depth analysis. We separated these studies into those that presented evidence from a pilot introduction of the SDM in an LMIC community (13) and those that examined results of national scale-up efforts of the SDM (22). In terms of study methodologies, all 13 pilot studies followed study cohorts for a period of 6–18 months, collecting user data at multiple points. None of the studies used a control or comparison group. The 22 national scale-up reports were cross-sectional implementation studies.

For pilot studies, our analysis focused on the following factors: (1) number of participants in study, (2) demographics of participants, (3) reasons for discontinuation of method, (4) approval of method, (5) number of participants who would recommend method, (6) ability to understand and use the method correctly, (7) previous experience with family planning, (8) intent to continue using the SDM, (9) reasons for using the SDM, (10) change to modern contraceptive prevalence rate (mCPR), (11) number of pregnancies, and (12) other outcomes of the SDM introduction.

For scale-up studies, our analysis used the following factors: (1) lead organizations in scale-up efforts, (2) whether the SDM was included in provider training, (3) if CycleBeads were in national procurement, (4) if the SDM was included in health management information systems or other national measurements and national family planning protocols, (5) number of service delivery points with the SDM, (6) number of service providers trained in the SDM, and (7) number of registered SDM users.

## RESULTS

### Pilot Introductions of the SDM

A total of 13 pilot studies to introduce the SDM were conducted in 10 countries: Albania, Benin, Burkina Faso, the Democratic Republic of the Congo (DRC), El Salvador, Ethiopia, Guatemala, India, Rwanda, and Turkey. Apart from Ethiopia, Guatemala, and Turkey, the remaining 7 pilot introductions and studies of the SDM were conducted by IRH with U.S. Agency for International Development funding. Although study methodologies varied slightly, each of these pilot studies involved first training health workers of various cadres including community health workers, health agents, nurses, midwives, and physicians to teach the SDM to new users with the assistance of CycleBeads, job aids, and visual brochures. Health workers were trained to counsel potential SDM users on the menstrual cycle, including the period start date and fertile window, and how to move the rubber ring along the different colored CycleBeads to track their cycle. On days 8–19, indicated by the white beads, users were taught to either abstain from sex or use a barrier method during intercourse. Users were taught the SDM in both home and clinic settings, and the SDM was often introduced with a range of other contraceptive methods and in routine family planning outreach and clinic settings. To accept the SDM, users were to report having regular cycles of 26–32 days. Those who accepted the SDM were offered the chance to participate in a study ranging from 6–18 months to follow their progress and experience using the method. In each study, recruitment of study participants was part of a program to introduce the SDM through family planning service delivery at home or in the clinic. Participation was wholly optional, and participants could have left the study at any time. Users did not have to participate in the studies to receive family planning services.

The pilot studies had sample sizes ranging from 76 to 767 users with a total of 2,906 users (Table 1). There was a wide range of study discontinuation rates, from 1%–45%, with a median of 30% discontinued method use within a 3–18 month period. The majority of users who discontinued use did so within the first 3–6 months. The primary reasons for method discontinuation were menstrual cycle lengths outside the range for SDM eligibility (419 users, 14%), unintended pregnancy (403 users, 14%), dissatisfaction with the method (125 users, 4%), and desire to become pregnant (107 users, 3%). Other reasons included unspecified personal reasons and switching to a different contraceptive method. The high numbers of users who discontinued because they had menstrual cycle lengths outside the range of eligibility demonstrated that many women may agree to use the SDM without having proper knowledge of their own history of cycle irregularity. This lack of knowledge could have been due to insufficient instruction by the health care provider or lack of previous tracking from the user, indicating a need for initial body literacy and fertility awareness instruction before introducing the SDM as a family planning method.

**TABLE 1.** Quantitative Results of Landscape Analysis of Pilot Studies in 10 Countries on the Standard Days Method of Family Planning

Study	Country	Participants, N	Discontinuation, N	Pregnancies, N	Approval, %	Would Recommend, %	Correct Use at 6 Months, %	Previous FP Use, %
Ram and Doracaj, 2007^7^	Albania	76	30	5		91	85	43^a^
Capo-Chichi and Anastasi, 2005^8^	Benin	219	33	21	90	90	95	45^a^; 39^b^; 20^c^; 7^d^
Bicaba et al., 2005^9^	Burkina Faso	79	20	2	90	90	95	22^e^
IRH, 2008^11^	Democratic Republic of the Congo	88	4	4	99	—	84	15^a^
IRH, 2005^14^	El Salvador	143	43	17	—	—	90	62^e^
Bekele, 2012^10^	Ethiopia	184	36	2	—	—	91	20^a^
Burkhart et al., 2000^13^	Guatemala	301	63	32	100	100^i^	95	88^a^
Dosajh, Ghosh, Lundgren, 2005^15^	India	230	82	20	99^f^^,^^h^; 70^g^^,^^h^	98^c^^,^^j^; 77^d^^,^^j^	87	74^f^^,^^k^; 66^m^^,^^p^; 1^n^^,^^o^^,^^p^
IRH, 2006^16^	India	482^q^; 285^r^	130^q^; 68^r^	77^q^; 20^r^	—	—	97	45^a^^,^^p^; 28^a^^,^^q^
Johri, Panwar, Lundgren, 2005^12^	India	482	225	73	90^s^	90^f^; 70^g^	98	59^t^; 41^c^; <3^p^
Blair et al., 2007^18^	Rwanda	121	30	16	—	—	99^f^; 88^g^	96^a^
Kalaca et al., 2005^6^	Turkey	132	53	4	—	—	—	—
Kursun, Cali, Sakarya, 2014^5^	Turkey	84	34	8	63^f^^,^^s^; 67^g^^,^^s^	—	—	12^n^

Abbreviations: FP, family planning; IRH, Institute for Reproductive Health.

a Never used modern method.

b Periodic abstinence.

c Condoms.

d Withdrawal.

e Not using modern family planning method in previous 2 months.

f Women.

g Men.

h Consider it a useful method.

i Of those who completed 1 year of use.

j Who hit 12 cycles.

k Had used some method in past, primarily condoms.

m Condoms.

n Withdrawal.

o Intrauterine device.

p Using method in previous 2 months.

q Rural.

r Urban.

s Satisfied with method.

t First-time family planning users.

Overall, the pregnancy rates among SDM users in the community studies ranged between 10% and 18% in each study population, which varied from the initial efficacy study that reported a 12% typical use pregnancy rate.^2^ This failure rate was calculated as percentage of users who experienced an unintended pregnancy while using the method over the study period, between 6 and 18 months dependent on the study. The different failure rates reported in the SDM pilot studies may indicate variability in the quality of the method's introduction, counseling, and screening of potential users. One study collated data from 1,646 users in 6 studies from various countries and reported a pregnancy rate of 14%, a figure closer to the rate found in the clinical trial.^4^ The majority of pregnancies occurred within the first 3 months of method use, which indicated users' failure to understand the method correctly, users' actual ineligibility for the method due to misunderstood patterns of irregular cycles, or husbands'/sexual partners' lack of cooperation to comply with the required abstinence/use of a barrier method on fertile days. This also indicated that the SDM had unique demand-side challenges as a family planning method, including a solid grounding in body literacy and fertility awareness and ability to communicate and negotiate sexual activity with a partner.

The trend in pregnancy rates matched results on correct use of the SDM among study participants. In most pilot studies, users were asked at multiple intervals to describe the mechanism of the SDM use, including how to identify the start of a menstrual cycle and manage fertile days. Answers to these questions improved over time. After 6 months of use, between 85%–99% of respondents correctly described the SDM, including which were the fertile days and how to correctly use CycleBeads, compared to 65%–75% in the initial surveys. This demonstrates how familiarity and comfort with the SDM, similar to other family planning methods, improved with time and use. Among users who continued the SDM beyond an initial 3 cycles, approval rates were very high, between 90%–100% saying they enjoyed the method and would recommend it to others. However, this approval figure was likely biased possibly from a courtesy bias dependent on the relation between study subject and data collector. In addition, these figures often did not include those users who discontinued the method in the initial months.

The demographics of users varied across study populations. Two studies found higher education levels among the SDM users compared to other family planning methods and the general population: in Turkey^5^^,^^6^ and in Albania, 49% of the SDM users had a high school degree, compared with 35% of users of other modern family planning methods.^7^ Four studies showed that the number of SDM users with a secondary education was higher than the general population: in Benin, 53% of SDM users had a secondary education^8^; in Burkina Faso, 64%^9^; in Ethiopia, 29%^10^; and in the DRC, 49%.^11^ More research is needed to understand why the method appealed to women with higher levels of education, but perhaps these women were better able to negotiate sex with partners, had more regular menstrual cycles due to better nutritional intake, or had more cognitive resources necessary to track the cycle.

Three studies measured the implementation of the SDM specifically in underserved, lower- educated populations. The majority of users in these studies had lower schooling levels or had never attended school. In rural Jharkhand State, India, 59% of SDM users had no schooling,^12^ in Guatemala, 32% of the SDM users were illiterate,^13^ and in El Salvador, 80% of the SDM users lived in rural areas and had less than a primary education.^14^

Both pregnancy rates and rates of correct use were similar between the higher-educated and lower-educated groups across all studies. Other demographic indicators, including parity, wealth, and place of residence, varied considerably and showed the wide range of users who were attracted to the SDM as a family planning method.

A consistent finding across the studies was reason for uptake of the SDM. The majority of users cited no side effects or health effects as the primary reason for choosing the method, while others cited low cost, no need for health visits, convenience, or existing familiarity with periodic abstinence as a family planning method. Measurement of previous use of modern methods of contraception varied across studies. For those that asked about ever use of modern family planning, between 15%–96% of SDM users said this was the first time they were using a modern method of family planning. Other studies asked about contraceptive use in the 2 months before SDM uptake, and answers varied between 21%–62% of users stating they were not using contraception in the immediate past. Common methods used recently included withdrawal, condoms, rhythm, and periodic abstinence.

The majority of users chose SDM because it has no side effects or health effects.

Only 3 studies measured change to overall mCPR in target communities after the introduction of the SDM. In El Salvador, the mCPR increased from 45% to 58%, with 4% of new contraceptive users using the SDM.^14^ Three other studies from separate communities in India had similar results, with mCPR increasing 7%–8% overall, with 1% of women using the SDM.^12^^,^^15^^,^^16^

Lastly, many users in the community studies cited improved couple's communication, increased male involvement in family planning, and more consistent condom use as additional benefits of using the SDM. A study from Bihar, India, demonstrated that “couple-based fertility awareness education is effective in increasing demand for contraception and improving knowledge of fertility overall.”^17^ In the pilot studies that asked about changes to male involvement and couple's communication after the SDM introduction, nearly all participants reported an improvement in joint decision making and male involvement.^8^^–^^11^^,^^15^^,^^18^

Many users cited improved couple's communication and increased male involvement in family planning among the added benefits of using the SDM.

### National Scale-Up of the SDM

Sixteen countries had conducted some level of national standardization and institutionalization effort for the SDM (Table 2). National scale-up efforts took place largely from 2001–2013 as part of the U.S. Agency for International Development-funded AWARENESS Project, of which IRH was the prime implementing partner. Each of the 22 national scale-up studies included in this review were published by IRH; no studies by other organizations were found in the database searches. Nationalization efforts involved incorporating the SDM in health worker training materials, adding CycleBeads in national commodity procurement, including the SDM in national health information measurement services, and integrating the SDM into national family planning protocols and policies. In studies examining the results of scale-up efforts, IRH also tracked the number of service delivery points providing the SDM, number of registered SDM users, and number of service providers trained in the SDM.

**TABLE 2.** Status of Scale-Up of the Standard Days Method of Family Planning in 16 Implementation Countries

Country	SDM in Training	SDM in National Measurements	SDM in National Protocols	Service Delivery Points With SDM, N	Service Providers Trained in SDM, N	Registered SDM Users, N
Benin	Yes	Yes	Yes	150	Not recorded	10,500
Bolivia	Yes	No	Yes	277	2,100	14,000
Burkina Faso	Yes	Yes	Yes	57	287	5,000
DRC	Yes	Yes	Yes	749	600	Not recorded
Ecuador	Yes	Yes	Yes	11	Not recorded	Not recorded
Guatemala	Yes	Yes	Yes	305	2,200	13,000
Haiti	No	No	No	20	141	700
Honduras	Yes	Yes	Yes	183	950	2,211
India, Jharkhand State	Yes	Yes	Yes	1,900	15,000	Not recorded
Madagascar	Yes	Yes	Yes	218	427	1,210
Mali	Yes	Yes	Yes	Not recorded	14,200	2,000
Nicaragua	No	No	Yes	336	1,308	343
Peru	Yes	Yes	Yes	348	725	7,862
Philippines	Yes	Yes	Yes	125	489	8,000
Rwanda	Yes	Yes	Yes	717	7,000	6000
Senegal	No	No	No	58	1,219	Not recorded

Abbreviations: SDM, Standard Days Method.

Although IRH led the process for institutionalization of the SDM in all 16 countries, local civil society groups helped lead efforts in Latin American countries, including Bolivia,^19^ Ecuador,^20^ Guatemala,^21^^,^^22^ Honduras,^23^ Nicaragua,^24^ and Peru.^25^^,^^26^ International NGOs had more involvement in other countries, including Benin,^27^ Burkina Faso,^28^ the DRC,^22^^,^^29^ Haiti,^30^ Madagascar,^31^ Mali,^22^^,^^32^^,^^33^ the Philippines,^34^ Rwanda,^22^^,^^26^^,^^35^^,^^36^ and Senegal.^37^ Local faith-based organizations had an active role in Burkina Faso (Catholic Diocese), the DRC (Catholic Relief Services), and Senegal (ChildFund). IRH also led efforts on the SDM integration with the Jharkhand State MOH in India.^26^^,^^38^^,^^39^

The extent of the SDM scale-up varied considerably across the different country contexts. Some countries, including Bolivia, Haiti, Nicaragua, and Senegal, id not complete the full national scale-up because of lack of interest or capacity of both local MOHs and civil society partners. In Senegal, efforts were limited to only 2 years of pilot activities in training and initial service provision with the NGO, Tostan, but the MOH chose not to continue programming for the SDM after the pilot. The situation in Haiti was similar; although the government supported a 2-year pilot introduction, they did not continue supporting the method in national protocols, reporting mechanisms, or training manuals beyond the initial pilot. Reasons for discontinuing support of the SDM were limited capacity and resources at the level of the MOH, competing priorities in family planning/reproductive health, and limited political will within the MOH.

Some countries discontinued support of the SDM because of limited capacity and resources at the level of the MOH, competing priorities, and limited political will.

In Latin America, the SDM was first introduced in key target areas with low modern contraception uptake and high numbers of users of traditional family planning methods. Bolivia and Nicaragua limited the SDM to these targeted populations with high demand for the method, and Ecuador, Guatemala, Honduras, and Peru continued to expand the method. All of the Latin countries had included the SDM in national health worker training, measurements, and protocols and policies. Decisions to take the SDM beyond initial pilots were made based on both the capacity and the will of local partners and country MOHs.

Aside from Senegal, every other African country included in scale-up efforts had the SDM included in national training, measurements, and family planning protocols. The SDM had the greatest institutional reach in the DRC. IRH partnered with 26 local organizations in the DRC and included considerable investment in social marketing for the method through Population Services International and Catholic Relief Services. The role of faith-based providers in the DRC was critical to expanded uptake of the method, and the national MOH also recognized both the local demand for and added value of the method.

A study in Rwanda demonstrated that 87% of trained community health workers correctly screened clients for eligibility to use the SDM based on cycle lengths/history and 92% accurately explained how to use CycleBeads. Further, 89% of clients reported knowledge of all key steps in the SDM after being counseled by the community health worker.^40^ There was some evidence that improved job aids could have overcome clinical providers' oversight of community health worker SDM provision and counseling,^40^ while task shifting or sharing were important and effective tools to disseminate the SDM.

IRH used an approach in Madagascar that was similar to efforts in the DRC, forging partnerships with faith-based organizations to promote the SDM nationally. The MOH in Mali recognized the demand for natural methods in the country and had consistent MOH advocates for the method who promoted family planning programming. Mali's MOH also encouraged the strategy of task shifting in SDM provision, training 13,000 community health workers in SDM teaching and promotion in areas with low uptake and accessibility to other modern methods of family planning.

Due to the decentralized nature of health policy and service provision in India, IRH partnered directly with the of Jharkhand State MOH in promoting the SDM, concentrating first on half of the state's districts with the greatest need for family planning services. Jharkhand included the SDM in state training, measurement, and family planning policies, and within 11 years, 6% of registered family planning users in Jharkhand were using the SDM, and half of the state population had heard of the SDM as a method of family planning.^39^

No country managed to include CycleBeads in national commodity procurements as it was considered an unconventional medical commodity and supplied by only 1 U.S.-based company. Supplies of CycleBeads were purchased through international NGOs, faith-based organizations, or donor bilateral funding and were then distributed within private and public sector clinics. The lack of national procurement of CycleBeads limited the continued implementation of the SDM and remained the most common barrier to national institutionalization of the method. The free digital versions of CycleBeads, in the form of mobile device apps, may help countries overcome this barrier in the future.

No country managed to include CycleBeads in national commodity procurements.

## DISCUSSION

This analysis showed various enablers and barriers to the SDM's community-level introduction and national-level scale-up. Pilot introductions were largely successful in generating demand for and correct use of the SDM, especially among new users of family planning. At the national level, the scale-up of the SDM were predicated on strong levels of government cooperation and broad-based coalitions of partners and advocates for the method. As in the cases of Bolivia, Haiti, Nicaragua, and Senegal, even after a successful pilot introduction of the SDM, without sufficient levels of advocacy and cooperation from national actors, the SDM will not be scaled up beyond the pilot intervention sites. Other countries with a more robust institutionalization of the SDM, including Burkina Faso and the DRC, had both strong levels of support from national stakeholders and an active coalition of partners invested in its implementation.

The low percentage of SDM acceptors in comparison to other contraceptive methods may have been a barrier to stakeholders' investment in national implementation of the method. Although community pilots demonstrated a level of demand for the SDM across numerous demographic indicators, in those studies that measured the percentage of SDM users within the broader contraceptive method mix, the percentage of family planning acceptors who chose the SDM did not move beyond 1%–5%. Worldwide, about 4% of all couples of reproductive age have used fertility awareness-based methods.^41^

Results from this analysis demonstrated that demand for the SDM was especially pronounced in communities already practicing fertility awareness-based methods of family planning, including less effective methods like periodic abstinence and withdrawal. High acceptance rates in Burkina Faso, the DRC, and among Mayan communities in Guatemala were all due to existing traditions of periodic abstinence and demonstrated the potential to improve the efficacy of these methods in teaching the SDM. The higher demand for the SDM in certain populations may have been a reason that national governments chose a more limited implementation of the SDM among targeted groups, rather than a national, system-wide scale-up.

Results also demonstrated other community benefits to the SDM introduction, including improved couple's communication, dispelled myths on modern contraception, and strengthened family planning use more broadly. For many couples, the introduction of the SDM was the first opportunity to discuss family planning jointly and involve men in decision making on both sexual activity and contraceptive use.

To achieve these results, pilot introductions of the SDM relied on direct training of frontline health workers and often employed an aspect of social marketing in the community to encourage conversations and improve knowledge of family planning overall. The importance of high-quality health worker training was especially evident when examining both the results on discontinuation of the SDM as a family planning method and failure rate in the initial 3 months of use. Although the majority of discontinuation occurred in the first 3 months of method use, either due to irregular cycles or unintended pregnancy, discontinuation rates varied considerably across study locations, likely demonstrating variants in teaching the method and appropriately screening eligible users. It also underscored the need for accompanying social and behavior change communication programming, particularly around body literacy and fertility awareness, to ensure both health care providers and clients understand the cycle requirements for eligibility and to better prevent unintended pregnancies in the initial months of use.

Although there was skepticism that teaching the SDM was overly complicated and cumbersome for service providers, evidence from studies in this analysis demonstrated the opposite. There was evidence that various cadres of health workers, including at the community level, had the capacity to adequately screen potential users for method eligibility. If properly trained, followed with supportive supervision, and equipped with job aids and guidelines, multiple levels of health providers taught the SDM effectively, which allowed users to understand the mechanisms of use quickly, and correct use improved over time. However, health workers were known to underutilize evidence-based practice guidelines.^42^ Guidelines developed, especially at the national level, were often not widely disseminated, and both in-service training and supportive supervision of health workers remained costly investments for governments with resource constraints.^43^

If properly trained, health providers taught the SDM effectively, and correct use improved over time.

The SDM was never more popular than other family planning methods, but there was still a demand for the method and other clear benefits to its introduction to improve client satisfaction and prevent unintended pregnancy. Those most attracted to the method were often first-time or discontinued users of modern family planning methods, and there was potential to improve contraceptive use within these populations by introducing the SDM. Further, inclusion of the SDM in family planning programming had potential to improve couple's empowerment in reproductive decision making, sexual negotiation, and consistent condom use.

Including the SDM in family planning programming had potential to improve couple's empowerment in reproductive decision making, sexual negotiation, and consistent condom use.

Moving the SDM away from donor-supported pilots required both method champions at the national MOH level and buy-in from health care providers within the clinic and community. This proved challenging as some family planning/reproductive health commentators argued against promoting the SDM in family planning programming, stating that there wasn't sufficient evidence on the efficacy of the SDM to merit its promotion as a modern method of family planning.^44^ This debate and skepticism of the method affected the level of investment in the SDM among family planning donors and implementing partners and buy-in at the national and community level in LMICs. Even within the countries targeted for national scale-up by IRH, barriers to its continued implementation remained. Some of the access barriers related to procurement may have been overcome given the development of the iCycleBeads app.

### Limitations

In reviewing the published literature available to complete this landscape review, 2 main limitations on the data arose: the preponderance of gray literature and high level of influence of IRH on the SDM research. The majority of literature on the implementation of the SDM were not peer reviewed, gray literature reports from foreign assistance programs. These reports were also all published by IRH. Researchers at IRH had also published 5 of the 9 peer-reviewed articles included in the study; the 4 others were published by non-IRH-affiliated researchers in Ethiopia,^10^ Guatemala,^13^ and Turkey.^5^^,^^6^ Even within external database searches, the high imprint of IRH on published material on the SDM was noteworthy. This demonstrated not only a limited investment in the SDM's implementation in LMICs outside of IRH but also a potential for bias in published literature, given IRH's initial development in the method and interest in its proliferation.

## CONCLUSION

Pilot introductions of the SDM demonstrated that the method was acceptable, especially to users of other fertility awareness-based methods of family planning or those concerned about side effects of hormonal contraception. Many SDM acceptors were first-time users of modern family planning. Other community benefits of the SDM's introduction included improved couple's communication, male involvement in family planning, and increased contraceptive uptake overall. Both discontinuation and method failure rates varied across study sites, highlighting the importance of high-quality health worker training in teaching the method and screening potential users. Tendency to experience unintended pregnancies in the first 3 months of use also demonstrated a need for more demand-side interventions to teach fertility awareness and body literacy when introducing the method to users.

At the national level, multiple barriers to the SDM's implementation and scale-up remained. Although the SDM had been piloted in over 30 countries worldwide, 16 countries had undergone rigorous scale-up processes to mainstream the SDM within the broader family planning method mix. Twelve of those countries had included the SDM in national family planning protocols, measurement tools, and health worker training, but no country managed to get CycleBeads into national procurement.

National implementation of the SDM was predicated on strong local political will and a broad-based coalition of local advocacy partners coordinating the method's implementation. There was little evidence of national scale-up of the SDM beyond the 16 countries included in this study. Although pilot studies demonstrated the potential for the SDM to match unmet contraceptive needs in key populations, strengthen male involvement, and increase overall family planning uptake, both demand-side and institutional barriers to include the SDM in the family planning method mix persisted.

## Notes

**Peer Reviewed**

**First Published Online:** February 7, 2020
